# Delays in diagnosing pulmonary tuberculosis within a context of medium incidence, Medellín, Colombia, 2017: an operational research

**DOI:** 10.1186/s12889-020-08829-9

**Published:** 2020-05-24

**Authors:** Iader Rodríguez-Márquez, Fernando Montes, Luz D. Upegui, Nilton Montoya, Nelly E. Vargas, Abelardo Rojas, Gloria C. Valencia, Claudia M. Álvarez, Leonardo Uribe, Jesús Ochoa

**Affiliations:** 1 Epidemiology Research Group, National School of Public Health, Universidad de Antioquia, Medellín, Colombia; 2 Secretary of Health of Medellín, Medellín, Colombia; 3 Institute of Medical Psychology and Medical Sociology, University Hospital of RWTH Aachen, Aachen, Germany; 4 Agreement Secretaria de Salud de Medellín-E.S.E Metrosalud, Medellín, Colombia

**Keywords:** Quality improvement, Health service utilization, Epidemiological surveillance, Tuberculosis

## Abstract

**Background:**

Delay in tuberculosis (TB) diagnosis is one of the first obstacles for controlling the disease. Delays generate greater deterioration of the health of the patients and increase the possibilities of transmission and infection at home and in the community. The aim of the study was to identify profiles and individual variables associated with patient delays and health care system delays in patients with pulmonary tuberculosis (PTB) in Medellín, Colombia, a city that notifies 1400 new cases per year.

**Methods:**

A retrospective cohort study in adults with PTB was conducted from May to September of 2017. Sociodemographic, health care-seeking behaviour, and clinical variables were measured. The outcomes were patient delay and health care system delay. The data were obtained from records of the local TB program, and a questionnaire was applied by the health care team that performs routine field visits. Simple correspondence analysis was used to identify groups (profiles), and their characteristics. Cox’s proportional hazards model was carried out to identify the variables associated with the delays.

**Results:**

The study included 183 patients. The total delay median was 101 days (IQR: 64–163). Patient delay was of 35 days (IQR: 14–84), the profile with greater delay belonged to consumers of psychoactive substances. The health care system delay was of 27 days (IQR: 7–89), the attributes of the profile with greater delay were being a female, having more than two consultations before the diagnosis, and having prescribed antibiotics. Basic-medium educational level [HR_a_ = 0.69; 95% CI (0.49–0.97)] and having a TB home contact [HR_a_ = 0.68; 95% CI (0.48–0.96)] were associated with greater patient delay. Having negative acid-fast bacilli (AFB) smear [HR_a_ = 0.64; 95% CI (0.45–0.92)] and more than two consultations before the diagnosis [HR_a_ = 0.33; 95% CI (0.22–0.49)] was associated with greater health care system delay.

**Conclusions:**

Data from epidemiological surveillance allowed locating risk groups with delays in TB diagnosis which requires the prioritisation of the local TB control program to promote early detection and prevention of adverse outcomes.

## Background

Tuberculosis (TB) control programs require an analysis focused on field epidemiological surveillance that allows generating effective interventions for its prevention and care. Delay in TB diagnosis is one of the first obstacles for the control program and a very important public health problem in the world. Delay generates greater worsening of the health of the patient and increases the possibilities of transmission of *Mycobacterium tuberculosis* at home and in the community [1, 2].

Delays in diagnosis are associated with suboptimal quality of TB care [3]. Low detection of cases may be related to individual and/or organizational conditions. The individual conditions are given by the person with possible TB diagnosis who does not seek medical care, while the organizational conditions are related to non-detection of cases by the health care system. The individual conditions have to do with actions taken by the patient with presumed TB to relieve their symptoms; this is denominated health care-seeking behaviour (HCSB) [4]. Both conditions generate delays in the diagnosis.

In Colombia, an incidence rate of TB of 32 cases per 100,000 inhabitants was estimated for 2016 [5]. The annual incidence rate of TB in Medellín has historically ranged between 40.4 and 65.6 cases per 100,000 inhabitants [6–8]. A Colombian study indicated that the total delay of patients with TB for 2014 in the city was of 61 days (IQR 32–105) [9].

Vries S.G.*et**al.,* [10] argued that to ensure equitable access to TB care it is necessary to adapt the programs to specific risk groups. We could not find studies describing the attributes associated with delays in TB diagnosis for Colombia. Moreover, the aim of the study was to identify the sociodemographic and clinical profile and the HCSB associated with the patient delay (PD) and health care system delay (HSD) in patients with pulmonary tuberculosis (PTB). Additionally, we aimed to identify the factors associated with the delays to prioritize risk groups in the city of Medellín.

## Methods

### Study design

A retrospective cohort study was carried out in adults with a new diagnosis of PTB from May to September of 2017.

### Setting

Medellín (Antioquia) is located in central-western Colombia, and it represents a locality with a high number of cases of tuberculosis within the country. Activities of TB prevention, surveillance, and care in Colombia are conducted in a decentralized manner [11]. Through a cooperation agreement with the Secretary of Health of Medellín and State Social Enterprise (E.S.E), Metrosalud home visits are conducted by health workers for the identification and seeking of active TB cases. For the homeless, the city has social assistance programs that offer shelter and treatment to TB patients.

### Participants

We included patients over 18 years old residing in Medellín who accepted the field visit. The study excluded patients with some degree of disability that hindered communication, those deprived of their freedom, and the homeless who were not institutionalized.

### Variables and data sources

Some sociodemographic variables (age, gender, educational level, marital status, socioeconomic level, health insurance, work status, homeless, and health care worker), HCSB variables (first behaviour by the patient, medical consultations before the diagnosis, and conduct of the health care provider) and clinical variables (tobacco use, alcohol consumption, consumption of psychoactive substance, medical history for diabetes mellitus, haemoptysis, HIV serological status, AFB smear, close contact with a TB patients, and chest X-ray) were measured. The socioeconomic level was estimated as socioeconomic strata using the geographic location of the residency of each patient. The outcome variables were the patient delay (PD) and health system delay (HSD). The PD was defined as the time elapsed in days since the start of PTB cardinal symptoms until the first contact with a health care provider. The cardinal symptoms (the presence of any) were: cough, chest pain, fever, weight loss, or haemoptysis. For the homeless, the health care provider was a professional, technician, or technologist in health care, and for the rest of the population was a physician. The HSD was the time elapsed in days since the first contact with a health care provider until the start of treatment [12].

The variables of the HCSB, cardinal symptoms, dates of onset of symptoms, and contact with a health care provider were collected through a standardized closed questionnaire applied through a personal interview in the house of the patient by the health care team during the routine field visit in TB cases. The final questions of the questionnaire met the criteria of relevance and suitability by two expert judges. The health care team were trained as interviewers for the closed questionnaire, and a pilot test was performed. The questionnaire was applied to the residents of a homeless shelter who fulfilled the inclusion criteria. The remaining data were obtained from the records in the local TB program.

### Study size

An apriori sample size, 170 patients, was estimated as formulated by Hsieh F.Y and Lavori P.W [13] for Cox proportional hazards model for non-binary covariables using a 0.05 significance level, a statistical power of 80%, and a hazard ratio for TB diagnosis delay for age (age HR = 0.65) selected from a previous study [14]. Finally, 183 patients were included in the study as consecutive cases from the start date. This non-probability sampling was carried out due to time and personnel limitations to conduct the field visits.

### Statistical methods

The median (in days) and the interquartile range (IQR) of the PD and the HSD were estimated. Delays in days were analysed as time to the event. The variables complying with the Hosmer Lemeshow criterion (*p* < 0.25) and those defined by theoretical criterion (age and gender) were used for profiling. A simple correspondence analysis [15] was conducted to identify the attributes of the profiles of the PD and of the HSD, and perceptual maps were constructed to illustrate the profiles.

To explore the relationship between the variables with the PD and HSD, Cox’s proportional hazards models were used. Confounding variables were controlled in the analysis phase. The assumptions were verified, and the HR was estimated with a 95% confidence interval. The STATA 14 statistical package was used (StataCorp, College Station, TX, USA Serial: 301406305146).

## Results

**Fig. 1:**
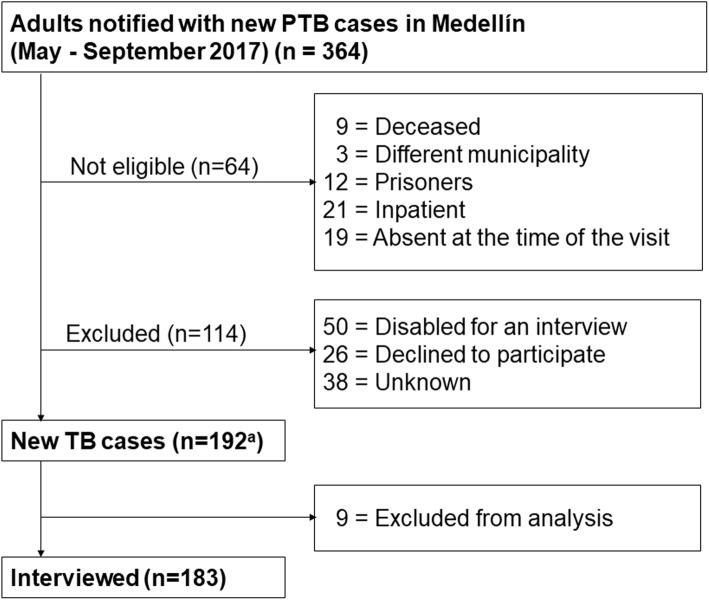
Flow diagram of the patient recruitment process. PTB: pulmonary tuberculosis. ^a^ Six homeless patients were included institutionalized in the municipal shelter

The study admitted 183 patients. Figure 1 illustrates the selection process. The median age of the not eligible and excluded patients was 42 years [IQR 27–62] and 69.7% thereof were male. The median age was 37 years [IQR 27–54]. The sex ratio was 0.9, and 66.67% of the patients had a basic-medium educational level. Most cases belonged to the low socioeconomic level (87.43%). Among the participants, 14% consumed some type of psychoactive substance (PAS), and coinfection with human immunodeficiency virus (HIV) was at 9.29%. One-third of the patients had negative sputum acid-fast bacilli (AFB). The first HCSB carried out was the consultation to a health care institution (40.98%), and the first conduct of the health care provider was the sputum AFB order (45.90%) (Tables 1, 2, and 3).

**Table 1:** Sociodemographic characteristics of new patients with pulmonary tuberculosis in Medellín, Colombia, May–September 2017

Sociodemographic characteristics	Total		PD		HSD	
	n (%)		Me [IQR]		Me [IQR]	
	183 (100)		35 [14–84]		27 [7–89]	
Age (years)^a^	37	[27–54]	–		–	
Age (years)						
< 60	149	(81.42)	35	[16–84]	24	[8–78]
≥ 60	34	(18.58)	39.5	[14–78]	32.5	[6–92]
Gender						
Female	96	(52.46)	30	[14–64]	32.5	[8–88]
Male	87	(47.54)	48	[15–91]	19	[7–91]
Educational level						
Basic-medium	122	(66.67)	48	[21–91]	25	[7–73]
Superior	61	(33.33)	28	[9–54]	33	[8–114]
Marital status						
Married-common-law	71	(38.80)	33	[14–65]	50	[11–113]
Single-separated-widowed	112	(61.20)	36.5	[16–92]	17	[7–63]
Socioeconomic level						
Medium-high	23	(12.57)	27	[7–41]	29	[13–93]
Low	160	(87.43)	39.5	[17–89]	26.5	[7–88]
Health insurance						
Subsidized	70	(38.25)	61	[30–106]	13	[5–37]
Others	113	(61.75)	29	[14–61]	45	[11–110]
Work status						
Unemployed	126	(68.85)	46.5	[19–90]	23	[7–92]
Employed	57	(31.15)	28	[12–61]	33	[11–73]
Homeless^b^						
No	177	(96.72)	35	[14–84]	29	[8–91]
Yes	6	(3.28)	63	[35–106]	7	[3–13]
Health worker						
No	172	(93.99)	36.5	[15–86]	27	[7–90]
Yes	11	(6.01)	21	[5–41]	20	[11–75]

*PD* patient delay; *HSD* health system delay; *Me* median; *IQR* interquartile range

^a^Median [interquartile range]

^b^Institutionalized in the municipal shelter

**Table 2:** Clinical characteristics of new patients with pulmonary tuberculosis in Medellín, Colombia, May–September 2017

Clinical characteristics	Total		PD		HSD	
	n (%)		Me [IQR]		Me [IQR]	
	183 (100)		35 [14–84]		27 [7–89]	
Tobacco						
No tobacco use	111	(60.66)	30	[14–91]	41	[11–106]
Past tobacco use	37	(20.21)	48	[9–75]	17	[6–62]
Current tobacco use	35	(19.13)	61	[28–87]	13	[4–51]
Alcohol consumption						
No	150	(81.97)	31	[14–74]	31	[8–92]
Former consumer	15	(8.20)	63	[19–101]	15	[6–50]
Currently	18	(9.83)	56	[15–106]	13	[7–67]
PAS consumption						
No	153	(83.61)	30	[14–65]	33	[9–93]
Former consumer	8	(4.37)	70	[20–178]	20.5	[6–35]
Currently	22	(12.02)	68.5	[40–127]	12.5	[3–30]
Medical history diabetes						
No	158	(86.34)	34	[14–84]	28	[9–92]
Yes	25	(13.66)	61	[25–91]	13	[4–64]
Haemoptysis						
No	147	(80.33)	35	[14–90]	26	[7–73]
Yes	36	(19.67)	34	[15–64]	34	[9–118]
HIV serological status						
Negative	166	(90.71)	35	[15–84]	26.5	[7–78]
Positive	17	(9.29)	35	[14–61]	33	[11–100]
AFB smear						
Positive	139	(75.96)	–		17	[7–63]
Negative	44	(24.04)	–		69	[27–130]
Close contact with TB patient						
No	139	(75.96)	31	[14–64]	32	[9–105]
Yes	44	(24.04)	63	[29–124]	15	[7–65]
Chest X-ray						
Yes	169	(92.35)	–		29	[7–89]
No	14	(7.65)	–		13.5	[13–29]

*PD* patient delay; *HSD* health system delay; *Me* median; *IQR* interquartile range; *PAS* psychoactive substances; *HIV* human immunodeficiency virus; *AFB* acid-fast bacilli

Note: The variables AFB smear and chest x-rays were not kept in mind for the PD

**Table 3:** Characteristics of the health care-seeking behaviour of new patients with pulmonary tuberculosis in Medellín, Colombia, May–September 2017

Characteristics of health care-seeking behaviour	Total		PD		HSD
	n (%)		Me [IQR]		Me [IQR]
	183 (100)		35 [14–84]		27 [7–89]
First behaviour conducted by the patient					
Consulted health institution	75	(40.98)	29	[7–75]	–
Self-medication	41	(22.40)	45	[28–84]	–
Took home remedies	36	(19.67)	48.5	[27–94]	–
Consulted private doctor	12	(6.56)	27.5	[8–71]	–
Consulted a pharmacy	9	(4.92)	59	[32–65]	–
Medical consultations before the diagnosis					
≤ 2	113	(61.75)	–	11	[5–30]
> 2	70	(38.25)	–	90	[45–145]
AFB smear as HCP conduct	84	(45.90)	–	13	[7–37]
Chest X-ray as HCP conduct	73	(39.89)	–	15	[6–59]
Antitussives as HCP conduct	46	(25.14)	–	70	[32–130]
Antibiotics as HCP conduct	29	(15.85)	–	63	[20–105]
Hx as HCP conduct	27	(14.75)	–	4	[2–12]
Analgesics as HCP conduct	7	(3.83)	–	65	[29–118]

*PD* patient delay; *HSD* health system delay; *Me* median; *IQR* interquartile range; *HCP* health care provider; *AFB* acid-fast bacilli; *Hx* hospitalization

Note: The variable first behaviour conducted by the patient was not kept in mind for the HSD. The variables medical consultations before the diagnosis, AFB as HCP conduct, chest X-ray as HCP conduct, antitussives as HCP conduct, and antibiotics as HCP conduct, Hx as HCP conduct and analgesics as HCP conduct were not kept in mind in the PD

**Fig. 2:**
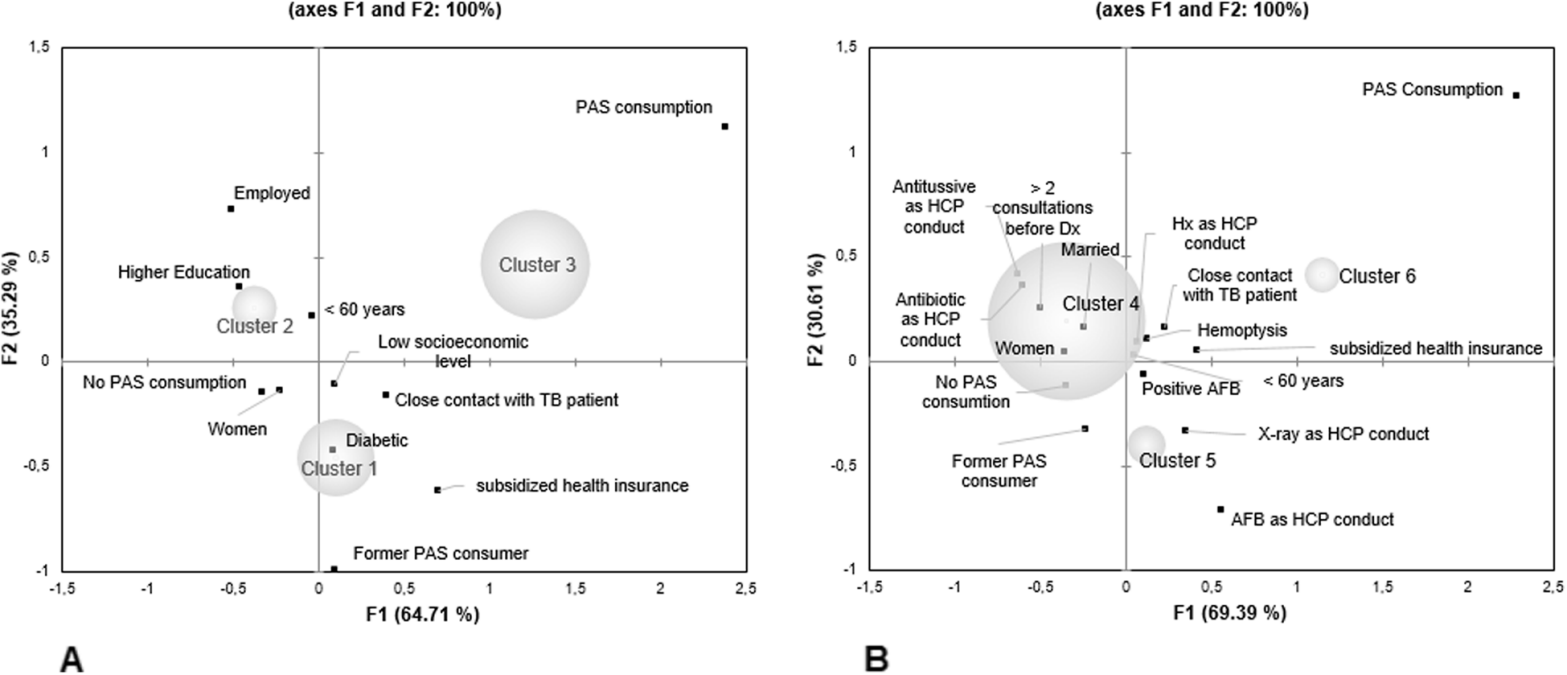
**a**. Profiles of patients with pulmonary tuberculosis related with the patient delay. **b**. Profiles of patients with pulmonary tuberculosis related with the health system delay. Dx: diagnosis; Hx: hospitalization; AFB: acid-fast bacilli; HCP: health care provider. Note: Shaded circles in the figure is a proportional representation of the median of the delay in each profile. PAS = psychoactive substances

The median of the total delay was of 101 days [IQR 54–163], where the PD was higher (Me = 35 days [IQR 14–84]) than the HSD (Me = 27 days [IQR 7–89]) (Supplementary file 1). Figure 2 illustrates the PD and HSD profiles (cluster). For the PD, profile one (Me = 50 days) was characterized for grouping individuals who were former PAS consumers, affiliated to the state-subsidized health insurance, with a history of diabetes mellitus, and close contact with a TB patient. Profile two (Me = 28 days) was defined by the presence of patients who were employed, with a superior educational level, and under 60 years of age. Lastly, profile three (Me = 74 days) was identified as having PAS consumers. Patients grouped in profile two had lower PD than those from profiles one and three (Kruskal-Wallis test, *p* = 0.0002).

Several profiles were identified in the HSD. Profile four (Me = 61.5 days) gathered female patients, who had more than two medical consultations before the diagnosis, and during their first contact with a health care provider were ordered antitussives or antibiotics. Profile five (Me = 13 days) was characterized by patients who during the first contact with the health care provider were ordered an AFB smear or a chest X-ray. Finally, profile six (Me = 12 days) was defined by the presence of PAS consumers. Patients placed in profiles five and six had lower HSD than those in profile four (Kruskal-Wallis test, *p* = 0.0001).

Tables 4 and 5 summarize the results of the Cox proportional hazards regression model for the PD and HSD. In the crude estimations, PD was longer for participants with a basic-medium educational level, subsidized health insurance, PAS consumption, and a close contact with a TB patient. DP was shorter in the health workers. The HSD was less extended in single people, subsidized health insurance, homeless, currently smoker, currently PAS consumer, close contact with a TB patient, BK as first contact with a health provider, Chest X-ray as the first contact with a health provider, and hospitalization. HSD was extended in patients with negative BK, more than two medical consultations before diagnosis, and antitussives as the first contact with a health provider.

**Table 4:** Characteristics associated with patient delay^a^

Characteristics	HR_**c**_ (95%CI)		Value ***p***	HR_**a**_ (95% CI)^**b**^		Value ***p***
Age (years)						
< 60	Ref.			Ref.		
≥ 60	1.08	(0.74–1.57)	0.681	1.09	(0.73–1.63)	0.679
Gender						
Male	Ref.			Ref.		
Female	1.27	(0.95–1.70)	0.110	1.09	(0.80–1.49)	0.568
Educational level						
Superior	Ref.			Ref.		
Basic-medium	0.63	(0.46–0.86)	0.003	0.69	(0.49–0.97)	0.034
Socioeconomic level						
Medium-high	Ref.			–	–	–
Low	0.73	(0.47–1.14)	0.169	–	–	–
Health insurance						
Other	Ref.			–	–	–
Subsidized	0.69	(0.51–0.94)	0.017	–	–	–
Work status						
Employed	Ref.			–	–	–
Unemployed	0.82	(0.60–1.13)	0.230	–	–	–
Health care worker						
No	Ref.			–	–	–
Yes	1.92	(1.04–3.56)	0.037	–	–	–
PAS Consumption						
No	Ref.			Ref.		
Former consumer	0.60	(0.29–1.22)	0.159	0.73	(0.35–1.54)	0.413
Currently	0.55	(0.35–0.87)	0.011	0.62	(0.38–1.01)	0.056
Medical history diabetes						
No	Ref.			–	–	–
Yes	0.69	(0.44–1.07)	0.095	–	–	–
Close contact with TB patient						
No	Ref.			Ref.		
Yes	0.66	(0.47–0.93)	0.019	0.68	(0.48–0.96)	0.030

*HR*_*c*_ crude hazard ratio; *95%CI* confidence interval at 95%; *HR*_*a*_ adjusted hazard ratio; *PAS* psychoactive substances; *AFB* acid-fast bacilli; *Dx* diagnosis; *HCP* health care provider; *Hx* hospitalization

^a^ The end point for patient delay was the first contact with a health care provider

^b^ Estimations adjusted for age, gender, educational level, PAS consumption, and close contact with TB patient

**Table 5:** Characteristics associated with the health system delay^a^

Characteristics	HR_**c**_ (95%CI)		Value ***p***	HR_**a**_ (95% CI)^**b**^		Value ***p***
Age (years)						
< 60	Ref.					
≥ 60	1.05	(0.72–1.52)	0.813	1.08	(0.72–1.62)	0.719
Gender						
Male	Ref.					
Female	0.84	(0.63–1.13)	0.251	1.00	(0.74–1.34)	0.976
Marital status						
Married - common-law	Ref.			–	–	–
Single-separated-widowed	1.52	(1.12–2.06)	0.008	–	–	–
Health insurance						
Other	Ref.			–	–	–
Subsidized	1.87	(1.37–2.54)	0.000	–	–	–
Homeless^c^						
No	Ref.			–	–	–
Yes	3.52	(1.53–8.13)	0.003	–	–	–
Tobacco						
No tobacco use	Ref.			–	–	–
Past tobacco use	1.45	(0.99–2.11)	0.051	–	–	–
Current tobacco use	1.81	(1.22–2.69)	0.003	–	–	–
PAS Consumption						
No	Ref.			–	–	–
Former consumer	1.34	(0.66–2.74)	0.423	–	–	–
Currently	1.84	(1.16–2.93)	0.010	–	–	–
Haemoptysis						
No	Ref.			–	–	–
Yes	0.73	(0.50–1.06)	0.103	–	–	–
AFB						
Positive	Ref.					
Negative	0.57	(0.40–0.81)	0.002	0.64	(0.45–0.92)	0.015
Close contact with TB patient						
No	Ref.			–	–	–
Yes	1.61	(1.13–2.28)	0.008	–	–	–
Medical consultations before Dx						
≤ 2	Ref.					
> 2	0.25	(0.18–0.36)	0.000	0.33	(0.22–0.49)	0.000
AFB as HCP conduct						
No	Ref.					
Yes	1.87	(1.38–2.53)	0.000	1.46	(1.05–2.02)	0.025
Chest X-ray as HCP conduct						
No	Ref.			–	–	–
Yes	1.48	(1.09–2.00)	0.011	–	–	–
Hx HCP conduct						
No	Ref.					
Yes	3.76	(2.43–5.84)	0.000	2.44	(1.53–3.89)	0.000
Antibiotics as HCP conduct						
No	Ref.			–	–	–
Yes	0.81	(0.54–1.21)	0.301	–	–	–
Antitussives as HCP conduct						
No	Ref.			–	–	–
Yes	0.57	(0.41–0.81)	0.001	–	–	–

*HR*_*c*_ crude hazard ratio; *95%CI* confidence interval at 95%; *HRa* adjusted hazard ratio; *PAS* psychoactive substances; *AFB* acid-fast bacilli; *Dx* diagnosis; *HCP* health care provider; *Hx* hospitalization

^a^ The end point for health system delay was the start of treatment

^b^ Estimations adjusted for age, gender, AFB smear, medical consultations before Dx, AFB smear as HCP conduct and Hx as HCP conduct

^c^ Institutionalized in municipal shelter

In the adjusted estimations, the educational level, the history of PAS consumption, and close contact with a TB patient were associated with the PD. Patients with a basic-medium educational level had a higher risk of prolonging the delay to the first contact with a health provider [HR_a_ = 0.69; 95% CI (0.49–0.97)]. PAS consumers had a more prolonged PD compared to non-consumers [HR_a_ = 0.62; 95% CI (0.38–1.31)]. Patients with positive close contact with a TB patient were late to consult with a health care provider [HR_a_ = 0.68; 95% CI (0.48–0.96)].

Having a negative AFB and more than two medical consultations before the diagnosis were associated with a greater HSD. The start of anti-tuberculosis treatment was “faster” in those who were hospitalized as initial conduct by the health provider [HR_a_ = 2.44; 95% CI (1.53–3.89)]. The instantaneous risk of starting anti-tuberculosis treatment was “faster” in those patients who were ordered an AFB by the health provider [HR_a_ = 1.46; 95% CI (1.05–2.02)].

## Discussion

This pioneering study in Colombia was conducted under routine programmatic conditions, given that it used data obtained during the visits to the house of the patients upon their diagnosis. The study was conducted with a population of patients with a high proportion of unemployment and living in the poorest socioeconomic levels of the city of Medellín, Colombia. Delays in TB presented a vast variability [16], where the PD was greater than the HSD. The profile with greater PD was comprised of PAS consumers, while the profile with lower PD was made up of economically active individuals with a superior educational level. The profile with greater HSD was constituted by patients with more than two medical consultations before the diagnosis and who were prescribed antitussive or antibiotic medications. Lack of clinical suspicion and paucibacillary forms of pulmonary TB have been associated with HSD [17]. Conducts such as hospitalization and ordering an AFB shortened the HSD.

Studies on factors associated with delays in TB are not consistent. This is mainly due to the variability of operational definitions (no consensus exists on an “acceptable” time for a delay) and to the diverse contexts of the health systems in each country [12, 16, 18, 19]. The literature has reported an association among lower educational levels and greater total delay [20, 21] and greater PD [14]. Few authors have explored the relationship between PAS consumption and the PD [22–24], as well as hospitalization and delays [25]. Similar results, Saifodine et al.*,* [26] found that positive close contact with a TB patient is associated with greater delay. Our finding of a higher number of consultations associated with the HSD is consistent with other studies [25–28]. Two authors also reported that a negative AFB status prolongs the HSD [26, 29].

Identifying patterns or profiles in epidemiology and public health by using correspondence analysis techniques have been scarce. This is a useful technique because it is reproducible and comprehensible by public health staff. This is the first study conducted on profiling the delay for a TB program in Colombia [15].

A study in Brazil analysed the performance of primary health services in TB diagnosis, locating a profile with lower delay constituted by patients who consulted specialized health services and had a lower number of medical consultations before the diagnosis [30]. The request for AFB or chest X-ray in the first medical consultation conformed a profile with a lower delay [31]. In profiling the HSD in this research, the number of consultations before the diagnosis and the prescription of antitussive and antibiotic medications could reflect the lack of clinical presumption.

The profile grouping economically active individuals with superior educational level was associated with a lower PD. This group has higher economic solvency and more knowledge about TB, which allows them to access health care services in a timely manner. The initial conduct by the health care provider of hospitalizing the patient accelerates the start of the treatment, which is explained by the opportunity of diagnostic tools in the institutions. However, hospitalizing the patient represents a financial burden for the health care system and has implications in the control of the institutional transmission of *M. tuberculosis*.

PAS consumers had a lower HSD, an apparently paradoxical finding; however, this might be attributed to a greater clinical presumption of TB by health professionals in some high-risk groups (stereotype). Homeless population have an additional care route for the diagnosis and start of treatment different from the rest of the population, mediated by government care programs and by altruism. Medellín has a network of day care centres for the homeless, which offers food, personal cleanliness, and provide medical care. When in contact with an individual with presumed TB, an AFB test is conducted, and if it is positive, the person is sought and offered to enter a shelter for treatment.

In contrast to the aforementioned, PAS consumption is associated with greater PD, coinciding with Belkina TV, Rabin AS, and Deponti GN [22–24]. This result could be explained by a lack of knowledge of the symptoms, attributing these to the consumption or to an inadequate diet that explains the weight loss characteristic of TB. Another possible explanation would be the fear of a possible hospitalization upon consulting a health care provider, a situation that would favour an abstinence syndrome by not being able to consume in the institution.

The study found a relationship between positive close contact with a TB patient and greater PD. Several explanations are suggested: those patients living with a sick person could show the shame of stigma, fear of the diagnosis and the rejection, and thus prolong the PD. Fear of the infection can exert a negative effect on the relationship between patients and health care providers [32].

No association was found among the socioeconomic level, work status, and the PD, although some studies have reported that low income could be associated with a greater PD [19, 33]. Agreeing with this work, Bloom BR [34] indicated that TB is also a problem of the health care system and not only a biological problem. Delays in the diagnosis due to ignorance of the cardinal symptoms of TB by service providers require educational interventions. Enhancement of health care systems, promotion of the diagnosis, and timely treatment in the risk groups identified in this study will allow the decrease of the delays and, in the long run, of the incidence in low- and medium-income countries.

Among the limitations of this study, it should be noted that only 50% of the new cases notified during the study period were included. Because of feasibility and availability of resources, the recruitment was done for only 4 months, and this could have affected the statistical power of the Cox regression model. However, no statistical difference was found between the participants and not eligible/excluded patients regarding age. The evaluation of the delays may have had memory bias given that the onset of symptoms were calculated based on the data collected from patients. The findings must be analysed with caution and cannot be generalizable for any other city, to patients with drug-resistant TB, minors, individuals deprived of their freedom, those with prolonged hospitalization, or those with some type of disability.

It is important to continue promoting the identification of risk profiles associated with delays in TB from a programmatic approach, which allows focalizing the interventions. Gupta R. K. et al., describe approaches for interventions aimed at homeless and PAS consumers. These interventions include detection tools and algorithms through active case search [35]. The research indicates that the TB control program in Medellín requires to improve the detection of cases in specific groups such as PAS consumers. For this purpose, it is necessary to come to an agreement – within medium-incidence contexts – as to what combination of diagnostic tests should be used in each risk group. It is important to implement strategies aimed at health professionals to improve their diagnostic presumption in TB. Another recommendation is to examine the route of care for the institutionalized homeless population to identify sub-processes that can be applied to lower HSD in the general population. Additionally, an agreement should be reached with the local TB control program on how to diminish the PD in the homeless population and PAS consumers.

In a broader context, the enhancement of TB control programs may require implementation research on the early recognition of TB symptoms. Studies are required to measure the PD and HSD in the inmate population and in patients with prolonged hospitalization.

## Conclusions

This study – frame-worked within the guidelines of operational research – [36] identified profiles of patients with PTB in Medellín, which are associated with the PD or the HSD. The total delay was prolonged (over 3 months). This finding must be discussed with those in charge of the local TB control program to prioritize interventions according to the local epidemiology of TB and to improve the quality of TB care in the city of Medellín. It is necessary to arrange for educational interventions aimed at health professionals, particularly regarding the best use of diagnostic tools and enhancing the active search for TB in PAS consumers, women, and in those with negative AFB during the first consultation.

## Supplementary information

**Additional file 1.** Supplementary file 1. Probability of having the first consultation with a health care provider and of starting treatment in new cases of pulmonary tuberculosis in Medellín, Colombia, May to September 2017.

## Data Availability

The datasets used and/or analysed during the current study are available from the corresponding author on reasonable request.

## Abbreviations

95% CI: Confidence Interval at 95%
AFB: Acid-fast Bacilli
Dx: Diagnosis
EFR: Epidemiological Field Research
HCP: Health Care Provider
HCSB: Health Care-Seeking behaviour
HIV: Human Immunodeficiency Virus
HR: Hazard Ratios
HRa: Adjusted Hazard Ratio
HRc: Crude Hazard Ratio
HSD: Health System Delay
Hx: Hospitalization
IQR: Interquartile Range
Me: Median
PAS: Psychoactive Substance
PD: Patient Delay
PTB: Pulmonary Tuberculosis
TB: Tuberculosis
