# Supplementary material for: Delays in diagnosing pulmonary tuberculosis within a context of medium incidence, Medellín, Colombia, 2017: an operational research
Source: BMC Public Health. 2020 May 24;20:757. doi: 10.1186/s12889-020-08829-9 (PMC7245903; doi:10.1186/s12889-020-08829-9)
Supplement: Supplementary file 1 — Additional file 1. Supplementary file 1. Probability of having the first consultation with a health care provider and of starting treatment in new cases of pulmonary tuberculosis in Medellín, Colombia, May to September 2017. [file 12889_2020_8829_MOESM1_ESM.docx]

**Supplementary material–Kaplan-Meir Analysis**

**Supplementary file 1.** Probability of having the first consultation with a health care provider and of starting treatment in new cases of pulmonary tuberculosis in Medellín, Colombia, May to September 2017 (n=183*)

| 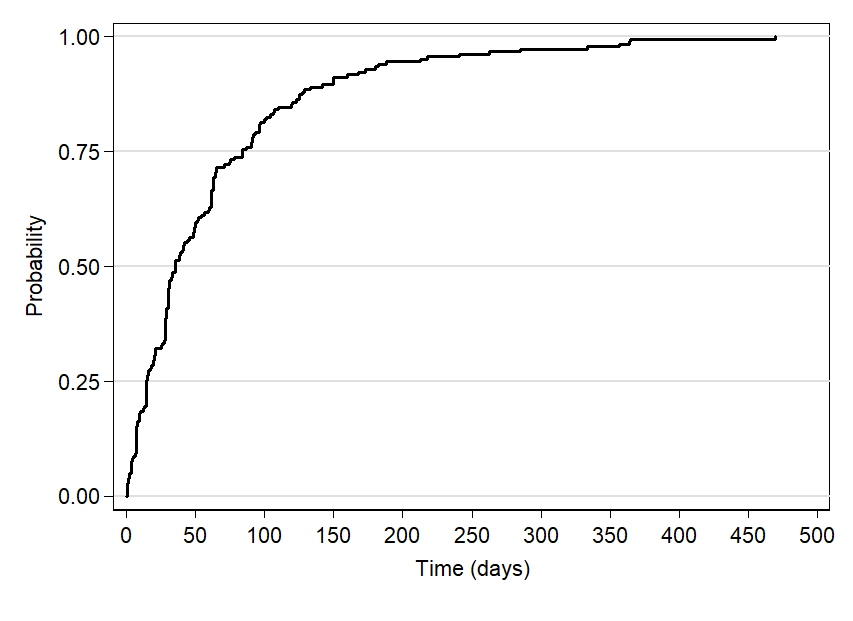 | 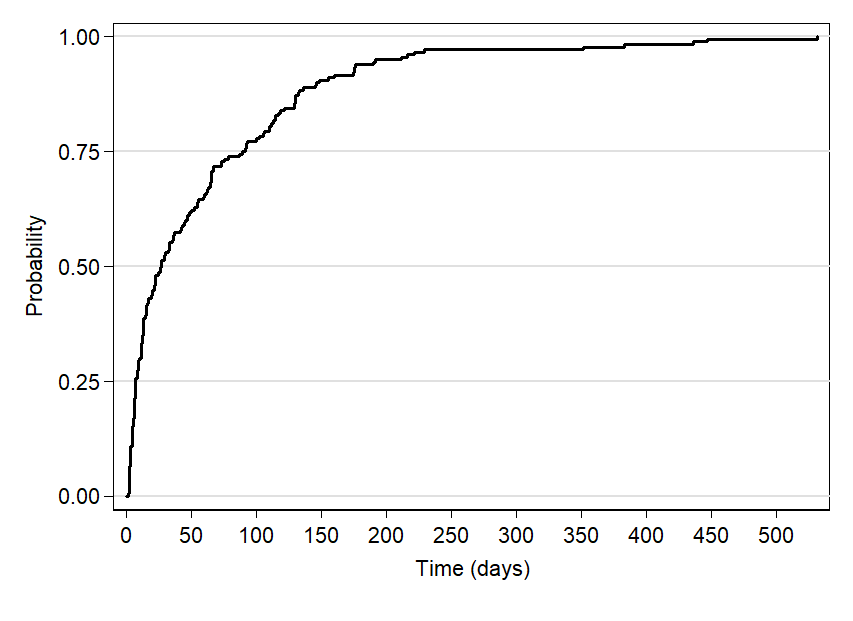 |
| --- | --- |
| a. Probability of having the first consultation with a health care provider (PD)** | b. Probability of starting treatment (HSD)*** |

* Nine cases with pulmonary tuberculosis without information regarding time at the first consultation with a health care provider and one case with unknown HIV status were excluded.

** Patient delay.

*** Health system delay.
